# [Retracted] Effects of the MAPK pathway and the expression of CAR in a murine model of viral myocarditis

**DOI:** 10.3892/etm.2025.12825

**Published:** 2025-02-17

**Authors:** Ling Niu, Chunli Li, Zhenzhou Wang, Hui Xu, Xinjiang An

Exp Ther Med 13:230–234, 2017; DOI: 10.3892/etm.2016.3909

Following the publication of the above paper, a concerned reader drew to the Editor's attention that, regarding the gel electrophoretic assay experiments shown in Fig. 1 on p. 231, unexpected areas of similarity were noted both between certain individual bands, and among the areas of the five sample lanes shown to represent the (A) control, (B) model and (C) intervention groups. Additionally, repetitive elements were noted in the corners of three of the panels showing the results of H&E staining of myocardial tissues in Fig. 5A-C on p. 233, which were difficult to attribute purely to coincidence.

Following an internal enquiry, the Editor of *Experimental and Therapeutic Medicine* has been able to verify the claims made by the interested reader; therefore, in view of the abovementioned anomalies that were identified, and owing to an overall lack of confidence in the presented data, the Editor has decided that the paper should be retracted from the Journal. The authors were asked for an explanation to account for these concerns, but the Editorial Office did not receive a reply. The Editor apologizes to the readership of the Journal for any inconvenience caused.

